# Efficacy of energy‐based devices on episiotomy pain and healing: A systematic review and meta‐analysis

**DOI:** 10.1002/ijgo.70764

**Published:** 2025-12-26

**Authors:** Shira Regev‐Sadeh, Chen Nahshon, Nadav Cohen, Ofer Lavie, Ariel Zilberlicht

**Affiliations:** ^1^ Department of Obstetrics and Gynecology, Carmel Medical Center Affiliated With the Rappaport Faculty of Medicine at Technion Israel Institute of Technology Haifa Israel; ^2^ Tel Aviv University Faculty of Medicine Tel Aviv Israel

**Keywords:** episiotomy, healing, infrared, low‐level laser, pain, post‐partum, visual analogue scale, wound

## Abstract

**Background:**

Episiotomy is a common obstetric procedure often associated with significant postpartum pain and delayed wound healing. Conventional treatments provide limited relief and might not be suitable for all women. Energy‐based therapies, including infrared irradiation and low‐level laser therapy (LLLT), a non‐thermal photo biomodulation technique, have shown potential for enhancing pain relief and tissue recovery, but their effectiveness in post‐episiotomy care remains unclear.

**Objective:**

This study evaluates the efficacy of energy‐based treatments on pain reduction and wound healing following episiotomy in postpartum women.

**Method:**

A database search was performed using MEDLINE with Ovid and PubMed interfaces, The Cochrane Central Register of Controlled Trials (CENTRAL), Embase, ClinicalTrials.gov and Web of Science up to December 2024. Prospective randomized and non‐randomized controlled trials were considered for inclusion. No restriction was imposed regarding the year or language of publication. Included studies compared one method of energy‐based treatment to placebo, standard of care, no treatment or another intervention. Data were synthesized using RevMan Web (Version 8.14.0) with a random‐effects model to account for interstudy heterogeneity. Pooled results were expressed as standardized mean differences (SMDs) with 95% confidence intervals. Evidence certainty was assessed using the GRADE approach.

**Results:**

A total of 173 studies were identified through database searches, of which 13 studies were included in the final analysis, encompassing a total of 1377 patients. Analyses were stratified by intervention type: infrared irradiation versus standard care or no treatment (*n* = 1088), LLLT versus placebo (*n* = 209) and LLLT versus therapeutic ultrasound (*n* = 80). Infrared irradiation reduced postpartum pain compared to standard care or no treatment (SMD = −0.50, 95% confidence interval [CI]: −0.98 to −0.02, *I* ^2^ = 88%, *P* < 0.01); however, it did not improve healing measures. LLLT showed no improvement in pain reduction (SMD: −0.31, 95% CI: −0.72 to 0.11, *I* ^2^ = 53%, *P* = 0.1) or healing (SMD: 0.23, 95% CI: −0.18 to 0.63, *I* ^2^ = 0%, *P* = 0.85) compared to placebo. LLLT compared to therapeutic ultrasound did not show advantage in pain or healing and was hindered by very high heterogeneity.

**Conclusion:**

Infrared therapy might reduce pain after episiotomy, although its effect on wound healing remains inconclusive. LLLT did not demonstrate significant benefits for pain relief or healing.

**Prospero Registration:**

CRD42024608543; registered December 2024.

## 1 INTRODUCTION

### 1.1 Background and rationale

Episiotomy is a common obstetric procedure involving an incision of the vaginal introitus during the second stage of labor to facilitate vaginal delivery.^1^ While episiotomy was widely practiced in the United States until 2006, the American College of Obstetricians and Gynecologists (ACOG) has since recommended against its routine use, instead endorsing a selective approach guided by maternal and fetal indications.^2^ As a result, episiotomy rates have declined substantially across most developed countries, estimated at below 10% in non‐operative vaginal deliveries.^3^, ^4^

Despite this overall decline, episiotomy continues to play an important role in contemporary obstetrics. When performed selectively, particularly mediolateral or lateral episiotomies in the setting of assisted vaginal births in nulliparous women, it has been shown to significantly reduce the risk of obstetrical anal sphincter injuries by 49%–68%.^5^ Thus, although no longer routine, episiotomy remains an essential intervention in specific clinical scenarios where maternal or fetal considerations warrant expedited birth or protection of the perineum. However, even when indicated and properly performed, episiotomy remains associated with several complications, including persistent perineal pain and delayed wound healing, both of which can adversely affect maternal quality of life. These complications have been linked to postpartum depression, difficulties in caring for the newborn, and delayed resumption of sexual activity.^6^

Management of post‐episiotomy pain and discomfort typically includes sitz baths, local perineal care, and topical agents.^7^ Current ACOG guidelines recommend a stepwise, multimodal approach to postpartum pain relief following vaginal delivery, beginning with non‐steroidal anti‐inflammatory drugs or acetaminophen and escalating to opioids when necessary.^8^ However, pharmacological treatments might be insufficient or undesirable for some women, highlighting the need for non‐pharmacological alternatives.

Energy‐based devices, including low‐level laser therapy (LLLT) and infrared irradiation, have been evaluated as potential therapeutic options for episiotomy pain and wound healing. These modalities are believed to enhance tissue repair, vasodilation, and microcirculation, mechanisms that might facilitate pain reduction and wound healing. Moreover, they have been successfully applied in the management of dermatologic, musculoskeletal, and other vulvovaginal conditions.^9^, ^10^, ^11^

### 1.2 Objectives

This systematic review and meta‐analysis aimed to evaluate the efficacy of energy‐based treatments, specifically LLLT and infrared irradiation, compared with placebo, standard care, no treatment, or other interventions in postpartum women who underwent episiotomy. The primary outcome was postpartum pain reduction, and the secondary outcome was improvement in wound healing. By synthesizing available evidence, this review seeks to clarify the clinical utility of these modalities in optimizing postpartum recovery and maternal well‐being.

## 2 METHODS

### 2.1 Eligibility criteria

Randomized controlled trials (RCTs) and prospective non‐randomized trials were considered for inclusion. No restriction was imposed on publication year or language. Eligible studies included postpartum women who underwent an episiotomy and received energy‐based treatments, including LLLT or infrared therapy, within 1 month of delivery. Comparators included placebo, standard of care, no treatment, or another intervention. Abstracts without a full‐text publication, observational studies, case reports, reviews, editorials, and non‐human studies were excluded. In cases of duplicate publications or overlapping populations, only the most recent or most comprehensive report was retained.

### 2.2 Information sources

A comprehensive literature search was performed by an experienced medical librarian using the following databases: MEDLINE (via Ovid and PubMed), The Cochrane Central Register of Controlled Trials (CENTRAL), Embase, ClinicalTrials.gov, and Web of Science. All databases were searched up to December 2024. A preliminary scoping search was conducted beforehand to confirm that no prior systematic review had specifically evaluated energy‐based treatments for episiotomy‐related pain or healing.

### 2.3 Search strategy

This systematic review and meta‐analysis was conducted according to the Preferred Reporting Items for Systematic Reviews and Meta‐Analyses (PRISMA) guidelines, and the review was structured using the PICO framework. The study protocol was prospectively registered at PROSPERO International Register of Systematic Reviews (CRD42024608543). Search terms and strategies are provided in Appendix S1.

### 2.4 Study selection

Titles and abstracts were independently screened by two researchers (e.g. S.R.S, A.Z.) using predefined eligibility criteria. Zotero software was used for reference management and duplicate removal. Non‐English abstracts identified during the search were translated by a professional translator; none met the criteria for full‐text review. All included full‐text studies were published in English.

Following title and abstract screening, all potentially eligible articles underwent full‐text evaluation by the same reviewers to determine final inclusion. Discrepancies were resolved through discussion with a third reviewer (C.N.).

### 2.5 Data extraction

Studies meeting all eligibility criteria proceeded to data extraction. This was performed independently by two reviewers using standardized tables. Extracted variables included study design, sample size, intervention characteristics, comparator, timing and duration of therapy, inclusion and exclusion criteria, outcome definitions, and effect measures. The primary outcomes were reduction in pain and improvement in healing, assessed using standardized clinical scales as defined in the original studies.

### 2.6 Assessment of risk of bias

The methodological quality of RCTs was assessed using the Cochrane Risk‐of‐Bias tool (RoB2),^12^ and non‐randomized studies were assessed using the Risk of Bias In Non‐randomized Studies of Interventions (ROBINS‐I) tool.^13^ The overall certainty of evidence for each outcome was evaluated according to the GRADE framework.^14^

### 2.7 Data synthesis

Analyses were conducted using Cochrane RevMan Web (Version 8.14.0) and the GRADEpro Guideline Development Tool. Methods followed the recommendations of Chapter 10 of the Cochrane Handbook for Systematic Reviews for Interventions.^15^ Standardized mean differences (SMD) were calculated due to variability in outcome scales across studies. All scales defined lower scores as indicating less pain or better healing.

Where the standard deviation of the change score was not reported, a shared standard deviation was calculated using a Pearson correlation coefficient of *r* = 0.5, consistent with Cochrane recommendations for situations where study‐specific correlations are unavailable.^16^ A two‐tailed *p*‐value <0.05 was considered statistically significant.

Heterogeneity was assessed using the *I* ^2^‐statistic. A random‐effects model was used due to clinical and methodological heterogeneity among the included studies. Publication bias was not assessed because each comparison included a limited number of studies. Subgroup analyses were performed for RCTs only versus all studies, by intervention type and by omitting one study at a time.

## 3 RESULTS

### 3.1 Study selection

**FIGURE 1:**
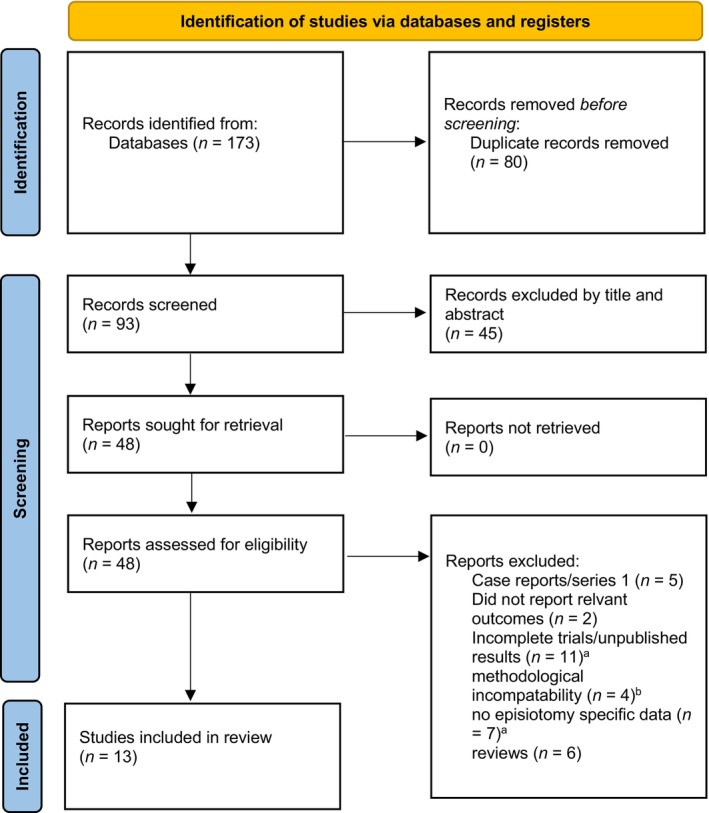
PRISMA flow diagram and article selection process. ^a^Data from studies with unpublished results or non‐episiotomy‐specific data were requested from authors but not received. ^b^No control groups or only post‐intervention pain/healing parameters reported. *Source*: Page et al.^40^

A total of 173 studies were identified through database searches, with 80 duplicates removed. The article selection process is detailed in Figure 1 and Table S1. After screening and eligibility assessment, 13 studies (10 RCTs and three non‐randomized prospective studies) were included in the final analysis.^17^, ^18^, ^19^, ^20^, ^21^, ^22^, ^23^, ^24^, ^25^, ^26^, ^27^, ^28^, ^29^

### 3.2 Study characteristics

The included studies comprised a total of 1377 postpartum women who had undergone episiotomy. Study characteristics are summarized in Tables 1 and 2.

**TABLE 1:** Study characteristics.

Study	Intervention	Total number of sessions	Session duration	Wavelength	Power/energy density	Device and manufacturer^a^
Infrared versus routine care or no treatment						
Gomathi et al. (2018)	Infrared lamp + routine care	6	NA	–	NA	Infrared lamp
Huang et al. (2019)	Far infrared radiation	5	40 min	–	160 W	Firapy WS Far infrared Therapy unit
Venkadalakshmi et al. (2010)	Infrared lamp	6	10 min	–	NA	Infrared lamp
Gayathri J et al. (2013)	Infrared lamp	NA	NA	–	NA	Infrared radiation
Sheoran et al. (2014)	Infrared light	6	15 min	–	NA	Infra red light
Ramesh et al. (2024)	Far infrared radiation	4	20 min	–	150 W	Murphy Far Infrared lamp
Li et al. (2021)	Infrared radiation + Mg sulfate compresses	6	30 min	–	NA	Infrared therapeutic apparatus (Beijing) Zhongke Zhaoyang Medical Technology Co. Ltd., no. 20172260575
Salvi et al. (2022)	Infrared radiation + routine care	6	15 min	–	150 W	Infrared light therapy
LLLT versus placebo						
Alvarenga et al. (2017)	Infrared LLLT	3	90 s	780 nm	5 J/cm^2^	portable Low‐Intensity Laser device, Twin LaserTM (MMOpticss Ltda, Brazil), classified as a class 3B laser with diode as the active element
Santos Jde et al. (2012)^b^	Red and infrared LLLT	1	30 s	660 nm 780 nm	8.8 J/cm^2^	portable clinical LLLT Twin LaserÒ (MMOptics, São Carlos, Brazil) Class 3B laser
Santos Jde et al. (2011)	Red LLLT	3	30 s	660 nm	3.8 J/cm^2^	portable LLLT Twin Laser (MMOptics®, Brazil), registered with the National Agency for Sanitary Surveillance (Number 80051420007) Class 3B laser
LLLT versus therapeutic US						
Chougala and Manishale (2015)	LLLT + cryo gel pad	3	30 s	660 nm	3.8 J/cm^2^	Low‐level laser therapy
Swaroopa and Deepthi (2017)	LLLT	NA	3 min	NA	2 J/cm^2^	Pulsed low‐level laser therapy

Abbreviation: LLLT, low‐level laser therapy; NA, not available; US, ultrasound.

^a^ As described in the methods section of each study.

^b^ The study had two interventional arms of red and infrared laser which were both compared to the control group. The control groups' participants were evenly split between the two comparisons, with the standard errors adjusted to account for the reduced sample size to prevent double‐counting.

**TABLE 2:** Study characteristics.

Study	Country	Design	Participants (*n*)	Time period of intervention post‐labor	Comparison	Timing of assessment^a^	Pain assessment scale	Healing assessment scale
Infrared versus routine care or no treatment								
Gomathi et al. (2018)	India	Prospective non‐randomized	460	3 days	Routine perineal care	4 days	VAS	REEDA
Huang et al. (2019)	Taiwan	RCT	78	3 days	No treatment	7 days	VAS	NA
Venkadalakshmi et al. (2010)	India	RCT	60	3 days	No treatment	3 days	VAS	NA
Gayathri J et al. (2013)	India	Prospective non‐randomized	40	3 days	Sitz Bath	3 days	NA	Southampton wound scale
Sheoran et al. (2014)	India	Prospective non‐randomized	60	3 days	Sitz Bath	3 days	NA	REEDA
Ramesh et al. (2024)	India	RCT	208	2.5 days	Sitz Bath	2 days	VAS	REEDA
Li et al. (2021)	China	RCT	120	3 days	Mg sulfate compresses	3 days	Dichotomous data yes/no improved	NA^b^
Salvi et al. (2022)	India	RCT	100	3 days	Cold compressions + routine care	3 days	NA	REEDA
LLLT versus placebo								
Alvarenga et al. (2017)	Brazil	RCT	43	2 days	Placebo	7–10 days	VAS	REEDA
Santos Jde et al. (2012)^c^	Brazil	RCT	114	2.5 days	Placebo	2.5 days	VAS	NA
Santos Jde et al. (2011)	Brazil	RCT	52	2 days	Placebo	2 days	VAS	REEDA
LLLT versus therapeutic US								
Chougala and Manishale (2015)	India	RCT	60	3 days	Therapeutic US + cryo gel pad	3 days	VAS^b^	REEDA^c^
Swaroopa and Deepthi (2017)	India	RCT	20	NA	Therapeutic US	7 days	NPRS	REEDA^c^

Abbreviations: NA, not available; NPRS, numeric pain rating scale; RCT, randomized‐controlled trial; REEDA, redness, edema ecchymosis, discharge and approximation of the wound edges scale; US, ultrasound; VAS, visual analogue scale.

^a^ Timing refers to post‐labor assessment points included in the meta‐analysis. Some studies reported additional assessment time points that were not included in the analysis.

^b^ Data was not included in the meta‐analysis due to missing baseline healing scores.

^c^ The study had two interventional arms of red and infrared laser which were both compared to the control group. The control groups' participants were evenly split between the two comparisons, with the standard errors adjusted to account for the reduced sample size to prevent double‐counting.

All participants were aged 18 years or older and received treatment within the first 10 days postpartum. Infrared therapy protocols typically involved applying repeated sessions within the first 3 days postpartum. LLLT interventions were delivered within the first postpartum week.

Pain outcomes were assessed at baseline and within 10 days postpartum, primarily using the visual analog scale. Two studies used alternative measures: the Numeric Pain Rating Scale and a dichotomous outcome (improved/not improved). Healing was generally assessed using the REEDA scale, except one study using the Southampton Wound Assessment Scale. One study did not report baseline healing and was therefore excluded from the healing meta‐analysis.

### 3.3 Risk of bias of included studies

**FIGURE 2:**
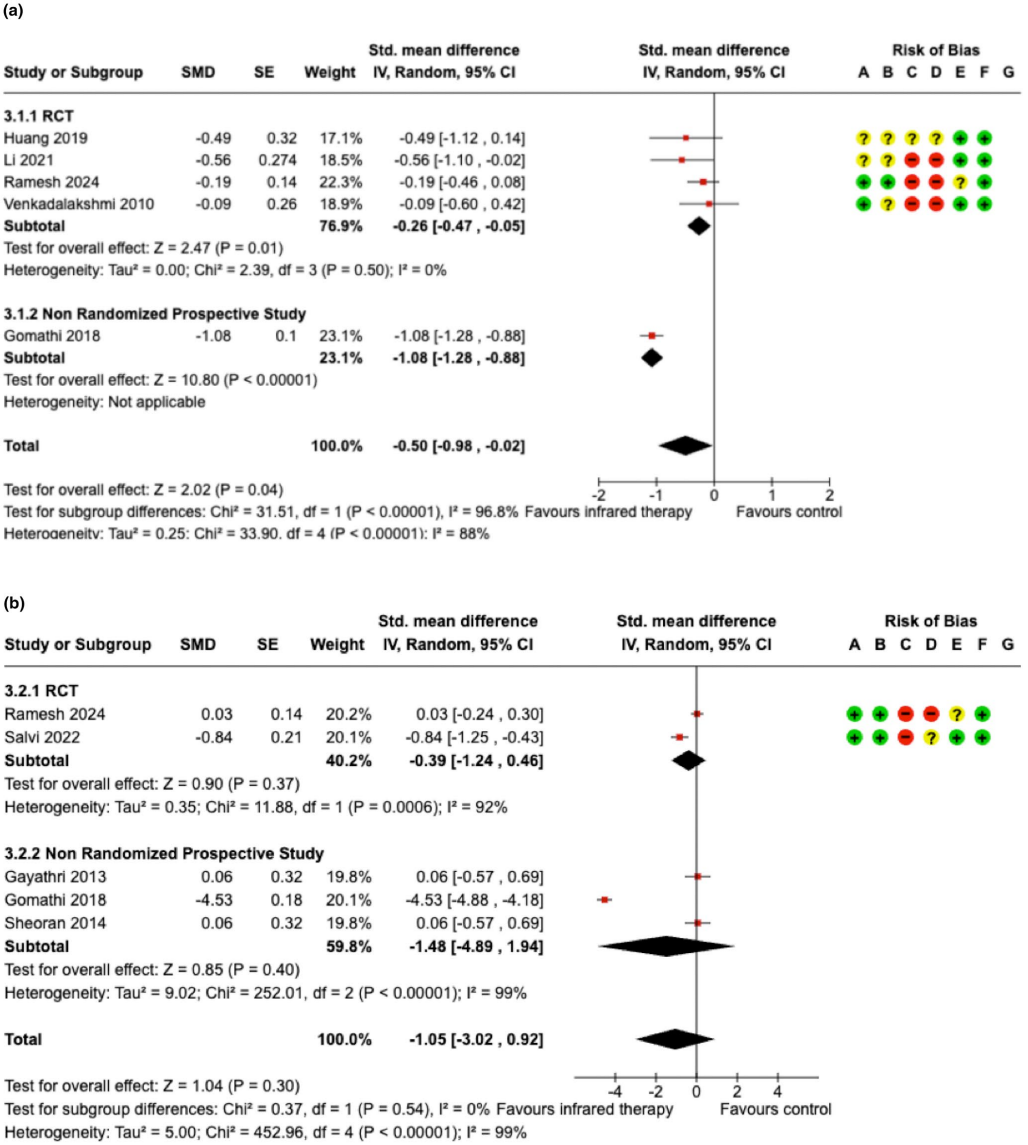
Infrared therapy compared to no treatment or standard of care. RCT, randomized‐controlled trial; SMD, standardized mean difference. (a) comparison of pain reduction; (b) comparison of healing measures; risk of bias legend: (A) random sequence generation (selection bias), (B) allocation concealment (selection bias), (C) blinding of participants and personnel (performance bias), (D) blinding of assessment (detection bias), (E) Incomplete outcome data (attrition bias), (F) selective reporting (reporting bias), and (G) other bias.

**FIGURE 3:**
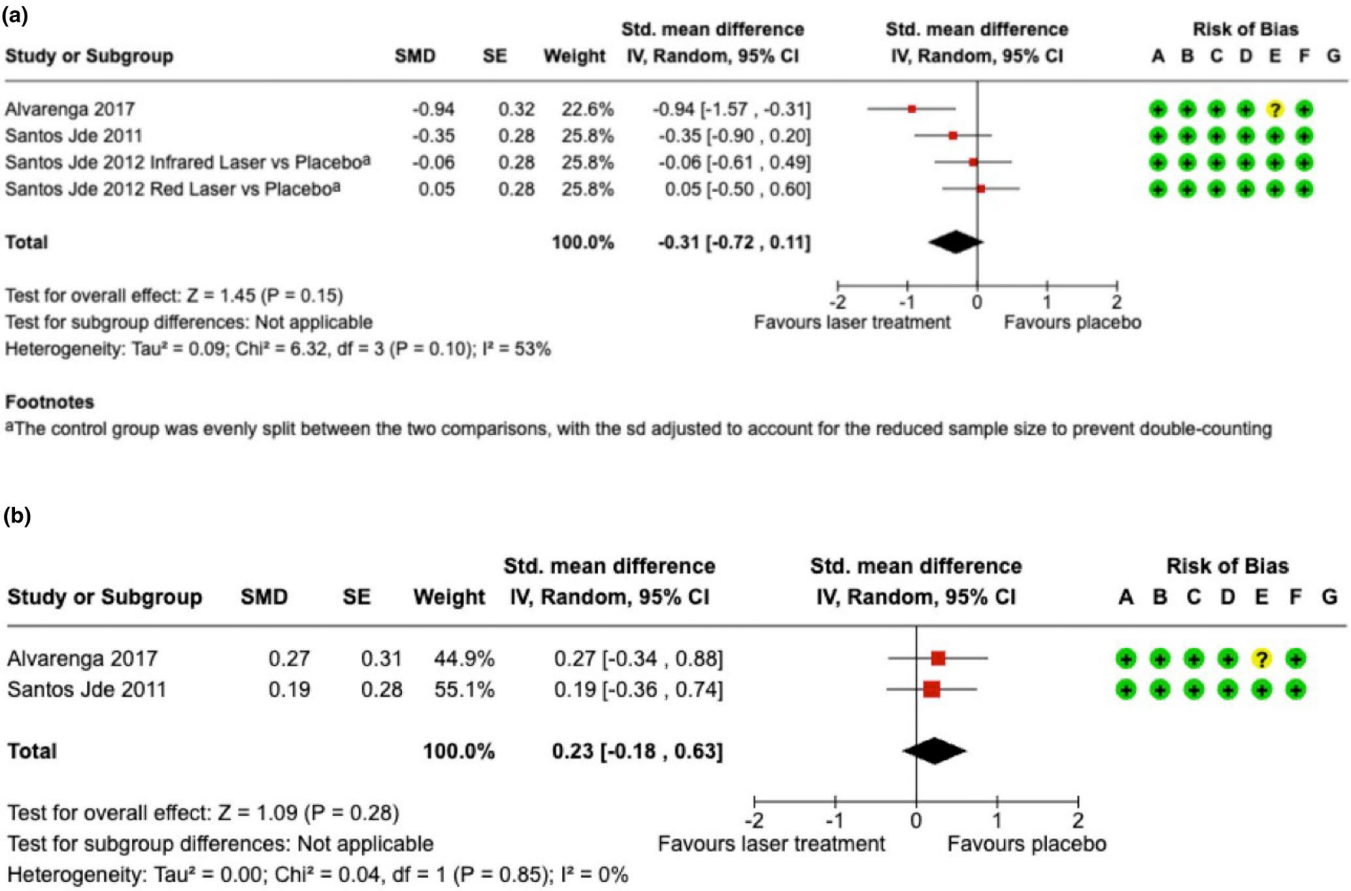
Low level laser therapy compared to placebo. SE, standard error; SMD, standardized mean difference. (a) Comparison of pain reduction; (b) Comparison of healing measures; risk of bias legend: (A) random sequence generation (selection bias), (B) allocation concealment (selection bias), (C) blinding of participants and personnel (performance bias), (D) blinding of assessment (detection bias), (E) incomplete outcome data (attrition bias), (F) selective reporting (reporting bias), and (G) other bias.

Risk‐of‐bias assessments for RCTs are shown in Figures 2 and 3 and Figure S1. Non‐randomized studies demonstrated moderate to serious risk of bias using ROBINS‐I (Table S2).

### 3.4 Synthesis of results

#### 3.4.1 Infrared irradiation

Eight studies (five RCTs and three non‐randomized prospective trials) evaluated infrared therapy versus routine care or no treatment, including 542 intervention participants and 546 controls.^20^, ^21^, ^22^, ^23^, ^25^, ^26^, ^27^, ^29^

Infrared therapy reduced postpartum pain compared with control across all studies (SMD = −0.50, 95% confidence interval [CI]: −0.98 to −0.02, *I* ^2^ = 88%, *p* < 0.01) and in RCTs alone (SMD = −0.26, 95% CI: −0.47 to −0.05, *I* ^2^ = 0%, *p* = 0.5). For healing outcomes, pooled SMDs did not demonstrate a statistically significant difference between infrared therapy and control and were characterized by substantial heterogeneity across all studies and in RCTs (Figure 2).

#### 3.4.2 Low‐level laser therapy versus placebo

Three RCTs evaluated the effects of LLLT versus placebo, including 124 women in LLLT groups and 85 controls. One study^18^ included three intervention arms, generating four pain comparisons. Only two studies contributed healing outcomes.^17^, ^19^

No statistically significant differences were observed for pain (SMD −0.31, 95% CI −0.72 to 0.11; *I* ^2^ = 53%, *p* = 0.10) or healing (SMD 0.23, 95% CI −0.18 to 0.63; *I* ^2^ = 0%, *p* = 0.85) (Figure 3).

#### 3.4.3 Low‐level laser therapy versus therapeutic ultrasound

Two RCTs compared LLLT with therapeutic ultrasound (US), each including 40 women per group.^24^, ^28^ These studies reported widely differing results for both pain and healing, with substantial heterogeneity in both magnitude and direction (Figure S1).

Sensitivity analyses indicated that no single study substantially altered results. Certainty of evidence ranged from moderate to very low (Appendices [Link], [Link], [Link]).

## 4 CONCLUSION

Infrared irradiation might reduce postpartum pain following episiotomy but did not significantly improve wound healing. LLLT did not demonstrate significant benefits compared with placebo.

### 4.1 Comparison with existing literature

While several systematic reviews have evaluated postpartum pain management strategies following perineal trauma, no previous meta‐analysis has specifically examined the role of energy‐based devices for episiotomy‐related pain and healing. A recent systematic review on acute pain management after vaginal delivery with perineal tears or episiotomy concluded that pain should be managed with acetaminophen, non‐steroidal anti‐inflammatory drugs, and cold therapy. While this review included one study comparing LLLT to therapeutic ultrasound, it did not assess infrared irradiation or other energy‐based devices.^30^ Similarly, another recently published meta‐analysis comparing various methods for pain relief and healing after perineal trauma found that cold application and perineal education were the most effective interventions. However, it included only one study on infrared irradiation and none on LLLT.^31^ A review by Choudhari et al., which focused specifically on episiotomy pain relief, concluded that infrared lamps and sitz baths are the two most effective and commonly used modalities for managing episiotomy‐related pain and healing.^7^ Despite these findings, to our knowledge, no previous meta‐analysis has specifically evaluated the effectiveness of energy‐based treatments, including infrared therapy, LLLT, and focused US, for episiotomy pain and healing, and our current meta‐analysis is the first published on this issue.

The proposed mechanisms underlying the benefits of infrared therapy on pain and tissue healing include thermal effects and photobiomodulation. These mechanisms are thought to enhance tissue oxygenation and perfusion, reduce inflammation, and promote cellular repair.^32^, ^33^ In our meta‐analysis, we found a significant improvement in pain outcomes among patients treated with infrared therapy, but no significant improvement in healing measures. One possible explanation for this discrepancy might be the mechanism of action of infrared therapy. By increasing blood flow, reducing inflammation, and modulating nerve activity, infrared therapy might provide immediate pain relief but might not necessarily accelerate tissue healing in a measurable way within the studies' timeframes. All included studies assessed healing within 1 week postpartum. Given that tissue remodeling and full healing extend beyond this timeframe, it is possible that longer follow‐ups are needed to capture the true impact of infrared therapy on wound healing.^34^

Low‐level laser therapy is increasingly used for tissue repair through non‐thermal photobiomodulation, operating within the visible to near‐infrared spectrum (390–1100 nm). It is thought to enhance mitochondrial ATP production, regulate cell signaling, stimulate growth factor synthesis, and reduce oxidative stress, thus promoting healing and pain relief.^35^, ^36^ Unlike infrared therapy, which mainly exerts its effects through thermal mechanisms, LLLT functions via non‐thermal photobiomodulation. Given their differing mechanisms, it is possible that infrared therapy provides more immediate analgesic effects, whereas LLLT might require prolonged or optimized dosing to influence tissue healing. Despite promising results in other fields, including musculoskeletal disorders, bone healing, and skin conditions,^9^, ^10^, ^37^ our meta‐analysis did not find a significant benefit of LLLT for episiotomy healing or pain relief. This might be due to small sample sizes limiting statistical power, suboptimal LLLT parameters in included studies, or fundamental differences in wound healing mechanisms in perineal tissue compared to other tissues where LLLT has shown efficacy. While our meta‐analysis did not demonstrate significant improvements with LLLT, a large retrospective study by Kymplova et al. reported significantly fewer and less severe complications in patients treated with laser therapy compared to controls (0.27% vs. 9.8%, *p* < 0.01). Although these findings suggest a potential benefit of LLLT, the retrospective nature of the study limits its comparability to our meta‐analysis, which included only prospective studies.

Only two studies in our meta‐analysis compared LLLT to therapeutic US, making it difficult to draw definitive conclusions due to small sample sizes and high heterogeneity. While both modalities are widely used for pain relief and tissue healing, their relative effectiveness in episiotomy recovery remains uncertain. Moreover, existing studies on pulsed US outside the context of perineal trauma have yielded inconclusive results regarding its therapeutic benefits.^38^, ^39^ Given the limited and inconsistent data, larger, well‐designed trials are necessary to determine whether one modality is superior or if their effects are comparable in this setting.

### 4.2 Strengths and limitations

This meta‐analysis has several strengths. It is the first to specifically evaluate the effectiveness of energy‐based treatments, including infrared therapy and LLLT, for episiotomy healing and pain relief. Thirteen prospective studies, including 10 RCTs, provided a reasonable evidence base. The literature search was comprehensive, and robust analytic methods were applied.

Several limitations should be acknowledged. Only 13 of the 173 screened studies met the inclusion criteria, raising the possibility of selection and publication bias. LLLT analyses were likely underpowered due to small sample sizes, potentially affecting the ability to detect significant effects. Considerable heterogeneity was present across studies, likely related to variations in treatment protocols, device types, outcome measurement instruments, and patient populations. Several studies did not report full details regarding device manufacturer or settings, representing an additional source of bias. None of the studies evaluating infrared therapy were blinded, increasing the risk of performance and detection bias.

Given these limitations, results should be interpreted with caution. Well‐powered, blinded RCTs with standardized methodologies are needed to better define the role of energy‐based therapies in episiotomy recovery.

### Implications

This meta‐analysis suggests that infrared therapy might reduce postpartum pain following episiotomy, whereas current evidence does not demonstrate significant benefits for LLLT for pain or wound healing. Effective management of postpartum pain remains essential for maternal well‐being, as persistent perineal pain and delayed recovery can affect mobility, newborn care, mental health, and sexual function. However, based on the limited and heterogeneous evidence available, energy‐based treatments cannot yet be recommended for routine clinical use. Future research should prioritize well‐designed, adequately powered randomized trials with standardized treatment parameters and longer follow‐up to determine whether these modalities have a meaningful role in postpartum recovery.

## AUTHOR CONTRIBUTIONS

**Shira Regev‐Sadeh:** manuscript writing, data analysis, data collection. **Chen Nahshon:** study design and planning, revision of manuscript. **Nadav Cohen:** revision of manuscript. **Ofer Lavie:** revision of manuscript. **Ariel Zilberlicht:** study design and planning, revision of manuscript, research supervisor.

## FUNDING INFORMATION

None.

## CONFLICT OF INTEREST STATEMENT

The authors have no conflicts of interest.

## Supporting information


Appendix S1.

Table S1.

Figure S1.

Table S2.

Table S3.

Appendix S2.

Appendix S3.

Appendix S4.

## Data Availability

Data sharing is not applicable to this article as no new data were created or analyzed in this study.
