# Supplementary material for: Efficacy of energy‐based devices on episiotomy pain and healing: A systematic review and meta‐analysis
Source: Int J Gynaecol Obstet. 2025 Dec 26;173(3):1284–94. doi: 10.1002/ijgo.70764 (PMC13173607; doi:10.1002/ijgo.70764)
Supplement: Supplementary file 1 — Appendix S1. [file IJGO-173-1284-s008.docx]

**Supplementary document S1: Search terms and strategies of database search**

**Ovid MEDLINE(R)**

1 exp Episiotomy/ 2545

2 "episiotom*".ab,kf,ti. 3190

3 1 or 2 3827

4 exp Laser Therapy/ 69095

5 exp Lasers/ 62255

6 exp Radiofrequency Therapy/ 45809

7 exp Infrared Rays/ 15686

8 (energy adj4 (transfer* or device* or based)).ab,kf,ti. 45373

9 "laser*".ab,kf,ti. 220868

10 ('radio frequency' or radiofrequency).ab,kf,ti. 43279

11 (infrared or 'infra red').ab,kf,ti. 127485

12 4 or 5 or 6 or 7 or 8 or 9 or 10 or 11 466434

13 3 and 12 23

**Embase:**

| **No.** | **Query** | **Results** | **Date** |
| --- | --- | --- | --- |
| **#16** | **#4 AND #15** | **53** | **27 Nov 2024** |
| **#15** | **#5 OR #6 OR #7 OR #8 OR #9 OR #10 OR #11 OR #12 OR #13 OR #14** | **776322** | **27 Nov 2024** |
| **#14** | **'far infrared radiation'/exp** | **21** | **27 Nov 2024** |
| **#13** | **'far infrared therapy'/exp** | **10** | **27 Nov 2024** |
| **#12** | **infrared:ab,kw,ti OR 'infra red':ab,kw,ti OR 'far infrared':ab,kw,ti** | **212883** | **27 Nov 2024** |
| **#11** | **'radio frequency':ab,kw,ti OR radiofrequency:ab,kw,ti** | **80530** | **27 Nov 2024** |
| **#10** | **laser*:ab,kw,ti** | **362141** | **27 Nov 2024** |
| **#9** | **(energy NEAR/4 (transfer OR device OR based)):ab,kw,ti** | **68753** | **27 Nov 2024** |
| **#8** | **'infrared radiation'/exp** | **50281** | **27 Nov 2024** |
| **#7** | **'radiofrequency therapy'/exp** | **52065** | **27 Nov 2024** |
| **#6** | **'laser'/exp** | **209159** | **27 Nov 2024** |
| **#5** | **'laser therapy'/exp** | **33941** | **27 Nov 2024** |
| **#4** | **#1 OR #2 OR #3** | **7100** | **27 Nov 2024** |
| **#3** | **episiotom*:ab,kw,ti** | **5049** | **27 Nov 2024** |
| **#2** | **'episiotomy complications'/exp** | **11** | **27 Nov 2024** |
| **#1** | **'episiotomy'/exp** | **6344** | **27 Nov 2024** |

**Clinical Trials.Gov:**

Total of 8 results

Searches:

Condition – Episiotomy

Intervention – 4 different searches:

1. Laser therapy – 4 results
2. 'radio frequency' or radiofrequency – 1 result
3. infrared or 'infra red' – 3 results
4. energy (transfer* or device* or based): no results

**The Cochrane Central Register of Controlled Trials (CENTRAL)** **16/12/2024**

ID Search Hits

#1 MeSH descriptor: [Episiotomy] explode all trees 402

#2 (episiotom*):ti,ab,kw 1440

#3 #1 OR #2 1440

#4 MeSH descriptor: [Laser Therapy] explode all trees 6173

#5 MeSH descriptor: [Lasers] explode all trees 3894

#6 MeSH descriptor: [Radiofrequency Therapy] explode all trees 2906

#7 MeSH descriptor: [Infrared Rays] explode all trees 275

#8 (energy NEAR/4 (transfer* or device* or based)):ti,ab,kw 1299

#9 (laser*):ti,ab,kw 26183

#10 ('radio frequency' OR radiofrequency):ti,ab,kw 6647

#11 (infrared OR 'infra red'):ti,ab,kw 6430

#12 {OR #4-#11} 40269

#13 #3 AND #12 38

**Web of Science 15/12/2024**

| # | Search Query | Database | Results |
| --- | --- | --- | --- |
| 1 | TS=(episiotom*) | All Databases | 5545 |
| 2 | TS=(laser*) | All Databases | 1795436 |
| 3 | TS=(energy NEAR/4 (transfer* OR device* OR based)) | All Databases | 689362 |
| 4 | TS=('radio frequency' OR radiofrequency) | All Databases | 518686 |
| 5 | TS=(infrared OR 'infra red') | All Databases | 1855572 |
| 6 | #5 OR #4 OR #3 OR #2 | All Databases | 4366068 |
| 7 | #1 AND #6 | All Databases | 50 |
