# Supplementary material for: Efficacy of energy‐based devices on episiotomy pain and healing: A systematic review and meta‐analysis
Source: Int J Gynaecol Obstet. 2025 Dec 26;173(3):1284–94. doi: 10.1002/ijgo.70764 (PMC13173607; doi:10.1002/ijgo.70764)
Supplement: Supplementary file 2 — Table S1. [file IJGO-173-1284-s007.docx]

Supplementary Table S2: Reasons for study exclusion at full-text evaluation stage

| **Reason for non-inclusion** | **References** |
| --- | --- |
| Case reports/series | [1–5] |
| No pain or healing measures reported | [6,7] |
| Incomplete trials/unpublished results | [8–18] |
| Methodological incompatibility (no control group, no baseline pain/healing scores) | [19–22] |
| No episiotomy specific data | [23–29] |
| Reviews | [30–35] |

1. Tamer Erel C, Urfalioglu M, Hamid R, Kargin OA, Ozcivit Erkan IB, Bayraktar E, et al. Ultrasonographic elastography, a new era showing the improvement of episiotomy scar treated with Er:YAG laser, a case-series. Lasers in surgery and medicine. 2024 Feb;56(2):127–32.

2. Safety and Efficacy of Er:YAG and Nd:YAG Laser Therapy in Gynecology: A Retrospective Case Series. 2019.

3. Cruz AR, Sales F, Chaves Carvalho J, Oliveira E, Agualusa L. PAIN INTERVENTIONAL TREATMENT AFTER EPISIOTOMY SENSITIZATION. Regional Anesthesia and Pain Medicine. 2022;47:A271.

4. Salim AS, Ahmed TM. KTP laser and fibrin glue for the treatment of vagino-perineal fistuale occuring after episiotomy. Annals of Saudi Medicine. 2002 Jul;22(3–4):262–4.

5. Erel CT. Case report: Vulvodynia treated with Erbium: YAG laser. EUROPEAN JOURNAL OF OBSTETRICS & GYNECOLOGY AND REPRODUCTIVE BIOLOGY. 2018 Dec;231:280–1.

6. Kymplová J, Navrátil L, Knízek J. Contribution of phototherapy to the treatment of episiotomies. Journal of clinical laser medicine & surgery. 2003;21(1):35–9.

7. Rzakulieva LM, Israfilbeili SG, Gasymova G. [Application of magnet laser radiation to stimulate healing of perineum injuries in the maternity patients]. Georgian medical news. 2006 Sep;(138):71–3.

8. R. B. R. zxb. Use of Infrared Laser in Vaginal Delivery. https://trialsearch.who.int/Trial2.aspx?TrialID=RBR-9zxb97. 2011;

9. Nct. Therapeutic Efficacy of Erbium: YAG Laser in Postpartum Patients With Episiotomy Scars. https://clinicaltrials.gov/ct2/show/NCT05793476. 2023;

10. Prospective Double Blind Controlled Trial of Low Level Laser Therapy - Cesarean Section or Vaginal Tear or Episiotomy. 2023.

11. Nct. MILTA vs Placebo Use Comparison for the Management of Pain Related to Perineal Scars Following Delivery. https://clinicaltrials.gov/show/NCT05345600. 2022;

12. Ctri. Episiotomy Wound Healing- comparison of infrared lamp therapy and kegal exercise. https://trialsearch.who.int/Trial2.aspx?TrialID=CTRI/2024/07/070743. 2024;

13. R. B. R. t8pw. Effect of Laser on maternal complications during the Postpartum period. https://trialsearch.who.int/Trial2.aspx?TrialID=RBR-3t8pw38. 2024;

14. Nct. Comparison Low-Level Laser Therapy With Cryotherapy in Parturients With Laceration and/or Episiotomy on Pain Reduction. https://clinicaltrials.gov/ct2/show/NCT06370910. 2024;

15. Nct. Comparative Effects of Cryotherapy and Infrared Light on Pain, Redness, and Healing of Episiotomy Wound. https://clinicaltrials.gov/ct2/show/NCT06325176. 2024;

16. Hafiza Neelam. Comparative Effect of Air Heat Versus Infra Red Heat on Pain and Wound Healling After Vaginal Delivery Episiotomy. https://clinicaltrials.gov/show/NCT05865236. 2023;

17. A Pilot Randomized Trial Comparing the Use of MILTA vs Placebo for the Management of Pain Related to Perineal Scars Following Delivery. 2022.

18. R. B. R. qm8jrp. Laser for pain relief in Nipple and Perineal Trauma in postpartum. https://trialsearch.who.int/Trial2.aspx?TrialID=RBR-2qm8jrp. 2023;

19. Farzana M, Shanmuga Priya R. Comparison of therapeutic ultrasound and low level laser therapy over pain and scar health in post episiotomy. Indian J Public Health Res Dev. 2020;11(2):113–8.

20. Boddupalli P. Use of infrared light fomentation for pain relief in postpartum mothers with episiotomy. Indian Journal of Public Health Research and Development. 2021;12(2):252–5.

21. Filippini M, Angioli R, Luvero D, Sammarini M, De Felice G, Latella S, et al. The Utility of CO2 Laser Treatment of Pelvic Symptoms in Women with Previous Perineal Trauma during Delivery. Journal of Personalized Medicine. 2024;14(1).

22. Novakov-Mikic A, Vizintin Z. LASER TREATMENT OF EPISIOTOMY - RELATED COMPLAINTS. Lasers in surgery and medicine. 2019 Feb;51:S39–S39.

23. Sierenska J, Sotomska Z, Madej-Lukasiak D, Waz P, Grzybowska ME. The Use of Capacitive and Resistive Energy Transfer in Postpartum Pain Management in Women after Perineal Trauma. Journal of Clinical Medicine. 2023;12(18).

24. Luvero D, Filippini M, Salvatore S, Pieralli A, Farinelli M, Angioli R. The beneficial effects of fractional CO2 laser treatment on perineal changes during puerperium and breastfeeding period: a multicentric study. LASERS IN MEDICAL SCIENCE. 2021;36(9):1837–43.

25. R. B. R. nwkfy. Photobiomodulation for pain relief, edema reduction and perineal laceration healing in the immediate postpartum period. https://trialsearch.who.int/Trial2.aspx?TrialID=RBR-103nwkfy. 2022;

26. Gondim EJL, Nascimento SL, Gaitero MVC, de Mira TAA, Goncalves A de V, Surita FG. Effectiveness of photobiomodulation therapy on pain intensity in postpartum women with nipple or perineal trauma: protocol for a multicentre, double-blinded, parallel-group, randomised controlled trial. BMJ Open. 2023 Dec;13(12).

27. Effect of Radiofrequency in the Treatment of de Novo Dyspareunia at 4 to 9 Months Postpartum: Randomised Prospective Trial. 2022.

28. Cristine Boniatti Constant É, Plentz Stein G, Camargo de Oliveira K, Laureano Paiva L, Martins Costa S, Geraldo Lopes Ramos J. Comparison of photobiomodulation with cryotherapy in the immediate postpartum period of parturients with grade I, grade II lacerations and/or episiotomy in reducing perineal and vulvar and edema: a randomized clinical trial. European journal of obstetrics, gynecology, and reproductive biology. 2024;301:240–5.

29. Bretelle F, Fabre C, Golka M, Pauly V, Roth B, Bechadergue V, et al. Capacitive-resistive radiofrequency therapy to treat postpartum perineal pain: a randomized study. PLoS One. 2020;15(4):e0231869.

30. Ezzati K, Fekrazad R, Raoufi Z. The Effects of Photobiomodulation Therapy on Post-Surgical Pain. JOURNAL OF LASERS IN MEDICAL SCIENCES. 2019;10(2):79–85.

31. Taha N, Daoud H, Malik T, Shettysowkoor J, Rahman S. The Effects of Low-Level Laser Therapy on Wound Healing and Pain Management in Skin Wounds: A Systematic Review and Meta-Analysis. Cureus. 2024 Oct;16(10):e72542–e72542.

32. Smith CA, Hill E, Denejkina A, Thornton C, Dahlen HG. The effectiveness and safety of complementary health approaches to managing postpartum pain: A systematic review and meta-analysis. Integr Med Res [Internet]. 2022;11(1). Available from: https://www.embase.com/search/results?subaction=viewrecord&id=L2014311210&from=export

33. Cobanoglu A, Sendir M. Evidence-Based Practices in Episiotomy Care. FLORENCE NIGHTINGALE JOURNAL OF NURSING-FLORENCE NIGHTINGALE HEMSIRELIK DERGISI. 2019 Feb;27(1):48–62.

34. Choudhari RG, Tayade SA, Venurkar SV, Deshpande VP. A Review of Episiotomy and Modalities for Relief of Episiotomy Pain. Cureus [Internet]. 2022 Nov 17 [cited 2024 Oct 27]; Available from: https://www.cureus.com/articles/110319-a-review-of-episiotomy-and-modalities-for-relief-of-episiotomy-pain

35. Girsang BM, Elfira E. A Literature Review on Postpartum Perineal Wound Care: Epidemiology, Impact, and Future Interventions. Open Access Macedonian Journal of Medical Sciences. 2023;11:73–80.
