# Supplementary material for: Efficacy of energy‐based devices on episiotomy pain and healing: A systematic review and meta‐analysis
Source: Int J Gynaecol Obstet. 2025 Dec 26;173(3):1284–94. doi: 10.1002/ijgo.70764 (PMC13173607; doi:10.1002/ijgo.70764)
Supplement: Supplementary file 3 — Figure S1. [file IJGO-173-1284-s001.docx]

Supplementary Figure 3 – Low level laser therapy compared to thearpeutic ultrasound


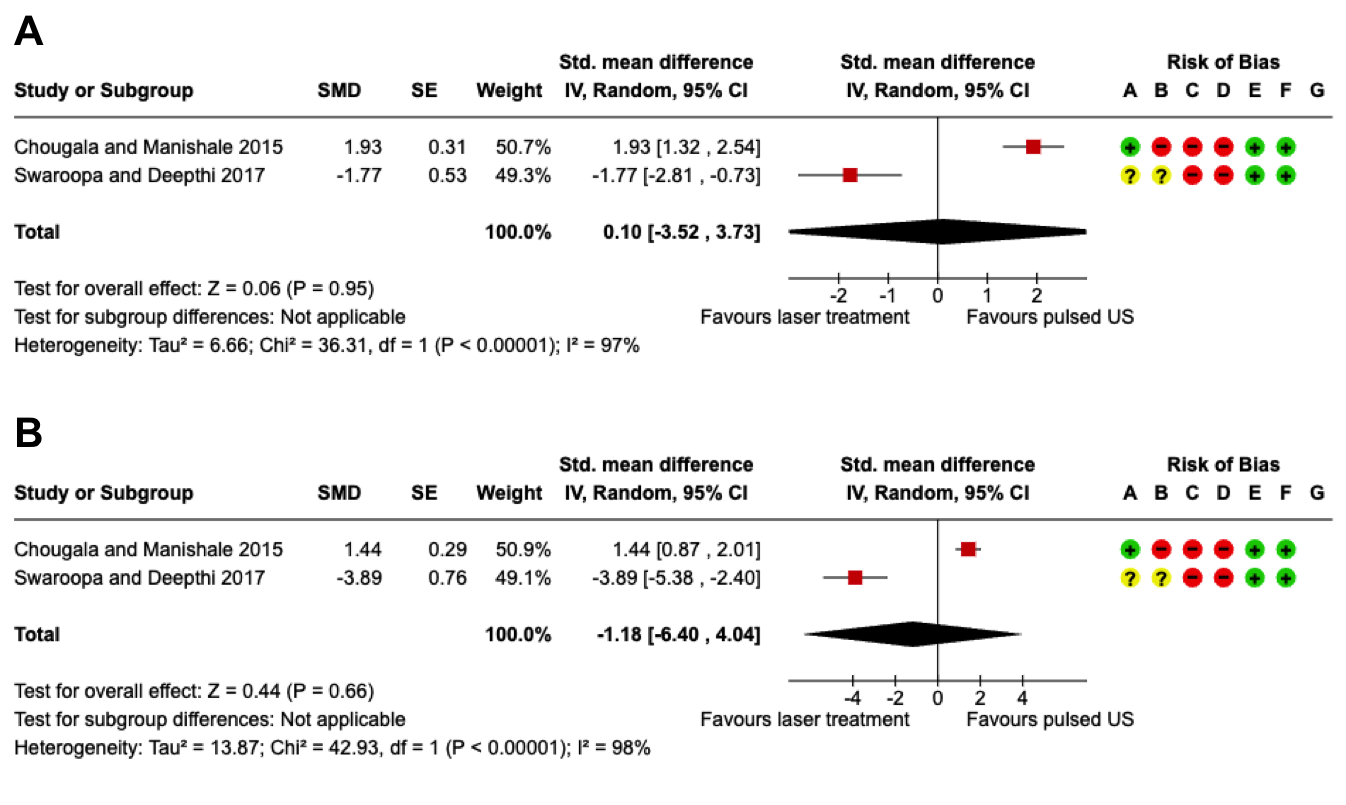


Abbreviations: SMD, standardized mean difference; SE, standard error

A, Comparison of pain reduciton B, Comparison of healing measures; Risk of bias legend: (A) Random sequence generation (selection bias), (B) Allocation concealment (selection bias), (C) Blinding of participants and personnel (performance bias), (D) Blinding of assessment (detection bias), (E) Incomplete outcome data (attrition bias), (F) Selective reporting (reporting bias), (G) Other bias
