# Supplementary material for: Efficacy of energy‐based devices on episiotomy pain and healing: A systematic review and meta‐analysis
Source: Int J Gynaecol Obstet. 2025 Dec 26;173(3):1284–94. doi: 10.1002/ijgo.70764 (PMC13173607; doi:10.1002/ijgo.70764)
Supplement: Supplementary file 4 — Table S2. [file IJGO-173-1284-s004.docx]

Supplementary Table S3: Quality Assessment of Prospective Non-Randomized Studies ROBINS-I

| **First author (year)** | **Pre-intervention domains** | | **At-intervention domains** | **Post-intervention domains** | | | | **Overall risk of bias** |
| --- | --- | --- | --- | --- | --- | --- | --- | --- |
|  | **Confounding** | **Selection bias** | **Information bias** | **Confounding** | **Selection bias** | **Information bias** | **Reporting bias** |  |
| Gomathi (2018) | Low | Low | Low | Low | Low | Moderate | Low | Moderate |
| Gayathri J. (2013) | Moderate | Moderate | Low | Low | Low | Moderate | Low | Serious |
| Sheoran (2014) | Low | Moderate | Low | Low | Low | Moderate | Low | Moderate |
