# Supplementary material for: Efficacy of energy‐based devices on episiotomy pain and healing: A systematic review and meta‐analysis
Source: Int J Gynaecol Obstet. 2025 Dec 26;173(3):1284–94. doi: 10.1002/ijgo.70764 (PMC13173607; doi:10.1002/ijgo.70764)
Supplement: Supplementary file 6 — Appendix S2. [file IJGO-173-1284-s005.pdf]

Summary of findings:

Infrared compared to standard of care or no treatment for episiotomy treatment to improve pain and healing measures

Patient or population: episiotomy treatment to improve pain and healing measures
Setting:
Intervention: infrared
Comparison: standard of care or no treatment

Outcomes	Anticipated absolute effects* (95% CI)		Relative effect (95% CI)	№ of participants (studies)	Certainty of the evidence (GRADE)	Comments
	Risk with standard of care or no treatment	Risk with infrared				
Pain reduction - RCT<br>follow-up: range 2 days to 7 days	-	SMD **0.26 lower**<br>(0.47 lower to 0.05 lower)	-	428<br>(4 RCTs)	⊕⊕⊕◯<br>Moderate[a]	Infrared treatment likely results in pain reduction
Pain reduction - total<br>follow-up: range 2 days to 7 days	-	SMD **0.5 lower**<br>(0.98 lower to 0.02 lower)	-	888<br>(5 non-randomised studies)	⊕◯◯◯<br>Very low[b,c]	Infrared treatment likely results in pain reduction
Healing improvement- RCT<br>follow-up: range 2 days to 3 days	-	SMD **0.39 lower**<br>(1.24 lower to 0.46 higher)	-	308<br>(2 RCTs)	⊕⊕◯◯<br>Low[c,d]	Infrared may have little to no effect on healing measures, but the evidence is very uncertain.
Healing improvement - total<br>follow-up: range 2 days to 4 days	-	SMD **1.05 lower**<br>(3.02 lower to 0.92 higher)	-	868<br>(5 non-randomised studies)	⊕◯◯◯<br>Very low[b,c]	Infrared may have little to no effect on healing measures, but the evidence is very uncertain.

***The risk in the intervention group** (and its 95% confidence interval) is based on the assumed risk in the comparison group and the **relative effect** of the intervention (and its 95% CI).

CI: confidence interval; **SMD:** standardised mean difference

GRADE Working Group grades of evidence
High certainty: we are very confident that the true effect lies close to that of the estimate of the effect.
Moderate certainty: we are moderately confident in the effect estimate: the true effect is likely to be close to the estimate of the effect, but there is a possibility that it is substantially different.
Low certainty: our confidence in the effect estimate is limited: the true effect may be substantially different from the estimate of the effect.
Very low certainty: we have very little confidence in the effect estimate: the true effect is likely to be substantially different from the estimate of effect.

Explanations

[a] Concerns regarding allocation concealment in several studies
[b] Non randomized trials included
[c] Large confidence intervals and statistical heterogeneity
[d] Visual inconsistency and statistical analysis showing heterogeneity
