# Supplementary material for: Efficacy of energy‐based devices on episiotomy pain and healing: A systematic review and meta‐analysis
Source: Int J Gynaecol Obstet. 2025 Dec 26;173(3):1284–94. doi: 10.1002/ijgo.70764 (PMC13173607; doi:10.1002/ijgo.70764)
Supplement: Supplementary file 7 — Appendix S3. [file IJGO-173-1284-s006.pdf]

Summary of findings:

LLLT compared to placebo for episiotomy treatment to improve pain and healing measures

Patient or population: episiotomy treatment to improve pain and healing measures
Setting:
Intervention: LLLT
Comparison: placebo

Outcomes	Anticipated absolute effects* (95% CI)		Relative effect (95% CI)	№ of participants (studies)	Certainty of the evidence (GRADE)	Comments
	**Risk with placebo**	**Risk with LLLT**				
Pain reduction follow-up: range 2 days to 10 days	-	SMD **0.31 SD lower** (0.72 lower to 0.11 higher)	-	209 (4 RCTs)	⊕⊕◯◯ Low[a,b]	LLLT may result in little to no difference in pain reduction, but the certainty of this evidence is low
Healing improvement follow-up: range 2 days to 10 days	-	SMD **0.23 higher** (0.18 lower to 0.63 higher)	-	95 (2 RCTs)	⊕⊕⊕◯ Moderate[c]	LLLT probably does not improve healing measures.

*__The risk in the intervention group__ (and its 95% confidence interval) is based on the assumed risk in the comparison group and the **relative effect** of the intervention (and its 95% CI).

CI: confidence interval; **SMD:** standardised mean difference

GRADE Working Group grades of evidence
High certainty: we are very confident that the true effect lies close to that of the estimate of the effect.
Moderate certainty: we are moderately confident in the effect estimate: the true effect is likely to be close to the estimate of the effect, but there is a possibility that it is substantially different.
Low certainty: our confidence in the effect estimate is limited: the true effect may be substantially different from the estimate of the effect.
Very low certainty: we have very little confidence in the effect estimate: the true effect is likely to be substantially different from the estimate of effect.

Explanations

[a] Visual inconsistency and statistical analysis showing heterogeneity
[b] Large confidence intervals and moderate number of participants
[c] Large confidence intervals and low number of participants
