# Supplementary material for: Efficacy of energy‐based devices on episiotomy pain and healing: A systematic review and meta‐analysis
Source: Int J Gynaecol Obstet. 2025 Dec 26;173(3):1284–94. doi: 10.1002/ijgo.70764 (PMC13173607; doi:10.1002/ijgo.70764)
Supplement: Supplementary file 8 — Appendix S4. [file IJGO-173-1284-s002.pdf]

Summary of findings:

LLLT compared to therapeutic US for episiotomy treatment to improve pain and healing measures

Patient or population: episiotomy treatment to improve pain and healing measures
Setting:
Intervention: LLLT
Comparison: therapeutic US

Outcomes	Anticipated absolute effects* (95% CI)		Relative effect (95% CI)	№ of participants (studies)	Certainty of the evidence (GRADE)	Comments
	Risk with therapeutic US	Risk with LLLT				
Pain reduction follow-up: range 3 days to 7 days	-	SMD **0.1 higher** (3.52 lower to 3.73 higher)	-	80 (2 RCTs)	⊕◯◯◯ Very low[a,b,c]	The evidence is very uncertain about the effect of LLLT on pain reduction when compared to pulsed US
Healing improvement follow-up: range 3 days to 7 days	-	SMD **1.18 lower** (6.4 lower to 4.04 higher)	-	80 (2 RCTs)	⊕◯◯◯ Very low[a,b,c]	The evidence is very uncertain about the effect of LLLT on healing improvement when compared to pulsed US

***The risk in the intervention group** (and its 95% confidence interval) is based on the assumed risk in the comparison group and the **relative effect** of the intervention (and its 95% CI).

CI: confidence interval; **SMD:** standardised mean difference

GRADE Working Group grades of evidence
High certainty: we are very confident that the true effect lies close to that of the estimate of the effect.
Moderate certainty: we are moderately confident in the effect estimate: the true effect is likely to be close to the estimate of the effect, but there is a possibility that it is substantially different.
Low certainty: our confidence in the effect estimate is limited: the true effect may be substantially different from the estimate of the effect.
Very low certainty: we have very little confidence in the effect estimate: the true effect is likely to be substantially different from the estimate of effect.

Explanations

[a] Concerns regarding allocation concealment
[b] Very high statistical heterogeneity
[c] Large confidence intervals and low number of participants
